# The genome and antigen proteome analysis of *Spiroplasma mirum*

**DOI:** 10.3389/fmicb.2022.996938

**Published:** 2022-11-02

**Authors:** Peng Liu, Yuxin Li, Youyuan Ye, Jiaxin Chen, Rong Li, Qinyi Zhang, Yuan Li, Wen Wang, Qingguo Meng, Jingyu Ou, Zhujun Yang, Wei Sun, Wei Gu

**Affiliations:** ^1^Hunan Provincial Key Laboratory for Special Pathogens Prevention and Control, Basic Medical School, Hengyang Medical School, Institute of Pathogenic Biology, University of South China, Hengyang, China; ^2^Key Laboratory for Aquatic Crustacean Diseases, College of Marine Science and Engineering, Nanjing Normal University, Nanjing, China; ^3^Jiangsu Provincial Center for Disease Control and Prevention, Nanjing, China

**Keywords:** genome, antigen proteome, *Spiroplasma mirum*, spiroplasma, pathogenesis

## Abstract

*Spiroplasma mirum*, small motile wall-less bacteria, was originally isolated from a rabbit tick and had the ability to infect newborn mice and caused cataracts. In this study, the whole genome and antigen proteins of *S. mirum* were comparative analyzed and investigated. Glycolysis, pentose phosphate pathway, arginine metabolism, nucleotide biosynthesis, and citrate fermentation were found in *S. mirum*, while trichloroacetic acid, fatty acids metabolism, phospholipid biosynthesis, terpenoid biosynthesis, lactose-specific PTS, and cofactors synthesis were completely absent. The Sec systems of *S. mirum* consist of SecA, SecE, SecDF, SecG, SecY, and YidC. Signal peptidase II was identified in *S. mirum*, but no signal peptidase I. The relative gene order in *S. mirum* is largely conserved. Genome analysis of available species in *Mollicutes* revealed that they shared only 84 proteins. *S. mirum* genome has 381 pseudogenes, accounting for 31.6% of total protein-coding genes. This is the evidence that spiroplasma genome is under an ongoing genome reduction. Immunoproteomics, a new scientific technique combining proteomics and immunological analytical methods, provided the direction of our research on *S. mirum*. We identified 49 proteins and 11 proteins (9 proteins in common) in *S. mirum* by anti-*S. mirum* serum and negative serum, respectively. Forty proteins in *S. mirum* were identified in relation to the virulence. All these proteins may play key roles in the pathogeny and can be used in the future for diagnoses and prevention.

## Introduction

*Spiroplasmas*, belonging to the class *Mollicutes* that includes *Mycoplasma*, *Phytoplasma*, and so on, featured by small genome sizes and a lack of peptidoglycan layer (Liu et al., 2018; Sasajima and Miyata, 2021). Spiroplasmas are geographically widespread as bacterial pathogens or symbionts in insects and plants (Liu P. et al., 2017) as well as important pathogens in commercially exploited aquatic crustaceans (Wang et al., 2004, 2005; Nunan et al., 2005). Spiroplasmas perform helical structure with well-defined cytoskeleton-like structure (Kürner et al., 2005; Trachtenberg, 2006) also show snake-like motility (Shaevitz et al., 2005) and unique infective characters including male-killing endosymbionts (Watts et al., 2009), which possibly involved in certain neurodegenerative diseases such as transmissible spongiform encephalopathies (Bastian et al., 2007). In recent years, this microorganism was studied as an attractive minimal model system (Trachtenberg, 2004), and its ultrastructural and macro-molecular characters have been well-described (Trachtenberg, 2004; Shaevitz et al., 2005; Trachtenberg et al., 2008).

Genome research was necessary and useful technique to investigate many species, it helps us to answer previously intractable biological questions (Sussman, 2015). Many bacterial whole genomes have been sequenced in the class of *Mollicutes* (including *Mycoplasma*, *Phytoplasma*, and *Spiroplasma*), the whole genome sequence of a few spiroplasma bacteria has yet to be published (Liu P. et al., 2017; Chen et al., 2019). Genomic research can provide us with more information on the characteristics of the morphology, structure, physiological metabolism, and genetic evolution of the bacteria (Chen et al., 2019). *Spiroplasma eriocheiris*, a pathogen of Chinese mitten crab *Eriocheir sinensis* and close to *Spiroplasma mirum* (Wang et al., 2011), performs chemotaxis without the conventional two-component system which was commonly found in bacterial chemotaxis. The cells are polarized by a tip structure and a dumbbell-shaped core in the tip connected by a fat ribbon, they are forming the internal structure of *S. eriocheiris*. The spiroplasma genomes reported so far do not have orthologs of other bacterial motility systems but have one tubulin homolog-FtsZ and five to seven MreB protein. MreB is related to actin, which is responsible for many eukaryotic motility systems (Takahashi et al., 2020). Sixteen proteins were identified as the components of the internal structure by mass spectrometry, including Fibril protein and four types of MreB proteins (Liu P. et al., 2017). MreB5, one of the five MreB paralogs, was proven the contributing to cell elongation and is essential for the transition from rod-to-helical shape in *Spiroplasma* (Harne et al., 2020). Cytoskeleton-like proteins (Fibril, Mrebs, and EF-Tu) were also phosphorylated suggesting that phosphorylation may play a crucial role in the formation of the cytoskeleton-like structure (Liu et al., 2020).

With the completion of a large number of biological genome sequences, while it is found that genome research alone does not explain the fundamental problem, because the ultimate function of various physiological functions in organisms is protein. Immunoproteomics as an emerging field of proteome analysis plays a key role in screening immunogenic proteins. Such a method has been used to successfully identify antigenic epitopes in several pathogens, such as *Mycoplasma bovis*, *Brucella abortus*, *Neisseria meningitidis*, and *S. eriocheiris* (Liu Y. et al., 2017).

Therefore, the study of proteomics has become the main research topic and direction in the post-genome era (Vandemoortele et al., 2016), and the most important issue for pathogenic microorganisms is to find the main immune proteins or antigen proteins (Aslam et al., 2017). That is to find the protein that plays a major role in the pathogenic process. The main method to study microbial immunoproteomics is to extract the whole protein or membrane protein of microorganism, electrophoresis, then hybridization with antiserum, so as to screen out antigen protein and then identify it by mass spectrometry or sequencing (Vandemoortele et al., 2016). Several mycoplasma immunoproteomics studies have been carried out using this method, such as *Mycoplasma genitalium*, *Mycoplasma pneumoniae*, *Mycoplasma hyopneumoniae*, *Mycoplasma synoviae*, and *Mycoplasma mycoides* (Khan et al., 2018).

*Spiroplasma mirum* originally isolated from a rabbit tick (*Haemaphysalis leporis-palustis* ATCC 29335 = CIP 103925) (Megraud et al., 1983). *S. mirum* and *S. eriocheiris are* close to each other in the phylogenetic tree and both can cause the cataract of neonatal rat (Hou et al., 2017). In this study, we mainly conducted comparative genomics studies on *S. mirum* and *S. eriocheiris* to find out the characteristics of the physiological metabolism, and genetic evolution of the spiroplasma, and to determine their different pathogenicity. We screened the antigenic proteins and provide a comprehensive view of the immunogenic proteins of *S. mirum* and also certainly provide valuable information for the identification of virulent proteins or diagnosis of pathogenic mechanisms.

## Materials and methods

### Bacterial strain

*Spiroplasma mirum*, Suckling Mouse Cataract Agent (SMCA) (Megraud et al., 1983), was originally isolated from rabbit ticks (*Haemaphysalis leporis-palustis*). We purchased SMCA [CIP 103925] from ATCC and cultured and cloned it with the same method (Itoh et al., 1989).

### Genome sequencing strategy

The complete genome sequences of *S. mirum* were firstly determined by high-throughput sequencing via pyrosequencing. A total of 170,589 sequences with an average length of 255 bp were obtained for the *S. mirum* genome, resulting in 37-fold genome coverage.

Assembly was performed using Newbler software of the 454-suite package, producing 19 contigs ranging from 0.5 to 490 kb for *S. mirum*.

Multiplex PCR was performed to determine the relationship of contigs, and closure of the gaps was performed by sequencing the PCR products. Phred, Phrap, and Consed software package^1^ was used for the final assembly and edition. Regions with poor sequencing quality and homopolymers were resequenced. The final consensus accuracy was 99.9999% for the *S. mirum* genome.

### Gene prediction and annotation

Putative protein-coding sequences (CDSs) were identified by Glimmer3 (Delcher et al., 1999) and ZCURVE 1.0 (Guo et al., 2003). Functional annotation of CDSs was performed through BLASTP searches against GenBank’s non-redundant (nr) protein database, followed by manual inspection. Transfer RNA genes were predicted by tRNAScan-SE (v1.23) (Lowe and Eddy, 1997). The metabolic pathways were constructed using KEGG database (Juncker et al., 2003; Kanehisa et al., 2004). Lipoprotein was determined by LipoP 1.0 (Juncker et al., 2003) and searching PROSITE motif PS51257. Protein domain prediction and COG assignment were performed by RPS-BLAST using NCBI CDD library.

An all-to-all BLASTP was applied to determine the paralogs. Two sequences in a pair are paralogs if the remaining HSPs cover at least 80% of the shorter protein’s length and if the identity is greater or equal to 50%.

### Comparative genomics

Orthologous proteins between *S. eriocheiris* and *S. mirum* were identified by all-vs-all reciprocal-BLASTP search, with criteria of similarity >20% and coverage >80%. Orthologs of known Mollicutes genomes were obtained from the MBGD database (Uchiyama, 2003). Unique genes were verified by TBLASTN search using protein sequences of each strain against the other strain’s genome sequence. Comparison of the gene order between *S. eriocheiris* and *S. mirum* was analyzed by Artemis Comparison Tool (ACT) (Carver et al., 2005). Protein synteny plot of *S. eriocheiris* and *S. mirum* was constructed by blast-score-ratio (Rasko et al., 2005).

### Identification of antigen proteins

The spiroplasma cells were activated in R2 medium to exponential phase and collected by centrifugation. After washing and resuspension, the sample was sonicated on ice and centrifuged to remove the cellular debris. Rabbit antiserum against *S. mirum* was prepared by following the methods described by Liu Y. et al. (2017). The serum was conjugated to Protein A Beads at 4°C for 4 h with gentle shaking. Conjugated antibody was added to the solution protein of spiroplasma and incubated at 4°C for 4 h. After a wash, the proteins were eluted by Glycine from the beads.

Dried protein pellets were reconstituted in 100 mM ammonium bicarbonate and protein concentrations were determined by Bio-Rad protein assay. Proteins were digested with trypsin and inactivated by heating at 99°C for 5 min. The solution was cleared by centrifugation and vacuum dried to remove bicarbonate salts.

Peptides were continuously separated by strong cation column (SCX) followed by C18 columns (Dionex) before being subjected to MS/MS analysis in an LTQ-Orbitrap mass spectrometer (Thermo Electron, Bremen, Germany). The mass spectrum data were initially searched against the database constructed using the predicted proteins of *S. mirum* with the aid of the Sequest search engine.

### Phylogenetic tree construction

We analyzed the genome of *S. mirum*, it revealed that *S. mirum* shares a total of 84 common proteins with *S. eriocheiris*, 14 Mycoplasmas, 4 Phytoplasmas, 3 Ureaplasmas, 1 Acholeplasma, and part of the genome sequence analysis of *Spiroplasma citri*. Concatenated protein sequences of 84 orthologous proteins of *mycoplasma* species were first aligned using MUSCLE (Edgar, 2004), then the conserved alignment blocks were extracted by the Gblocks program (Castresana, 2000). The phylogenetic tree was built using the maximum likelihood method implemented in PHYML (Guindon and Gascuel, 2003) using the following parameters: 100 replications for bootstrap analysis, “JTT” for substitution model, “estimated” for the proportion of invariable sites, “estimated” for gamma distribution parameters, “4” for the number of substitution categories, “yes” to optimize tree topology, and “BIONJ” for starting tree(s). Graphical representation and edition of the phylogenetic tree were performed with MEGA4.

### Nucleotide sequence accession number

The complete genome sequence and annotation of *S. mirum* were deposited in GenBank under accession numbers CP002082.

## Results

### Genome features

**FIGURE 1:**
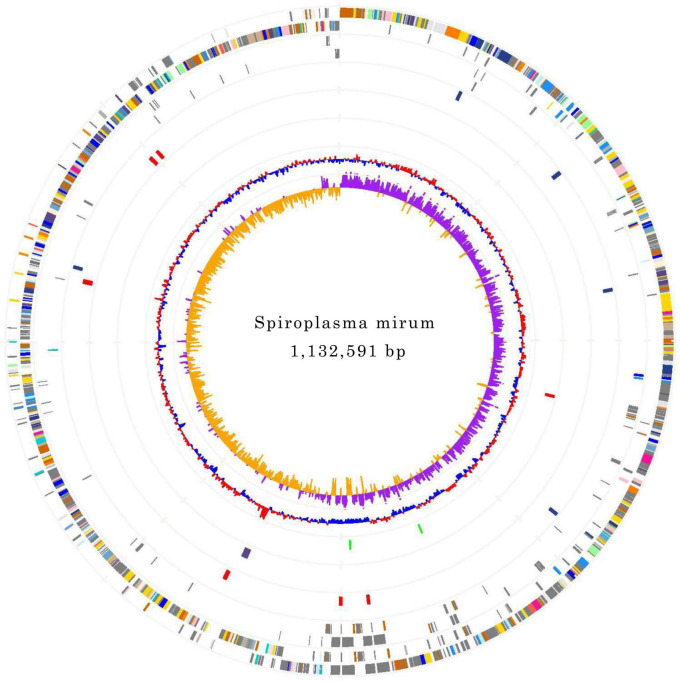
Chromosome Atlas of *Spiroplasma mirum*. Moving inside, each concentric circle represents a different genomic feature. The outermost circle shows predicted protein-coding sequences on both strands, colored by functional categories according to COG classification. The second circle displays specific genes of *S. mirum* genome compared with the other. The third circle illustrates tRNA genes on plus strand **(blue)** and minus strand **(red)**, and ribosomal RNA genes **(green)**. The fourth circle presents IS elements. The fifth circle shows mean centered G + C content (red-above mean, blue-below mean). The sixth circle **(innermost)** represents GC skew (G - C)/(G + C) calculated using a 1 kb window.

**FIGURE 2:**
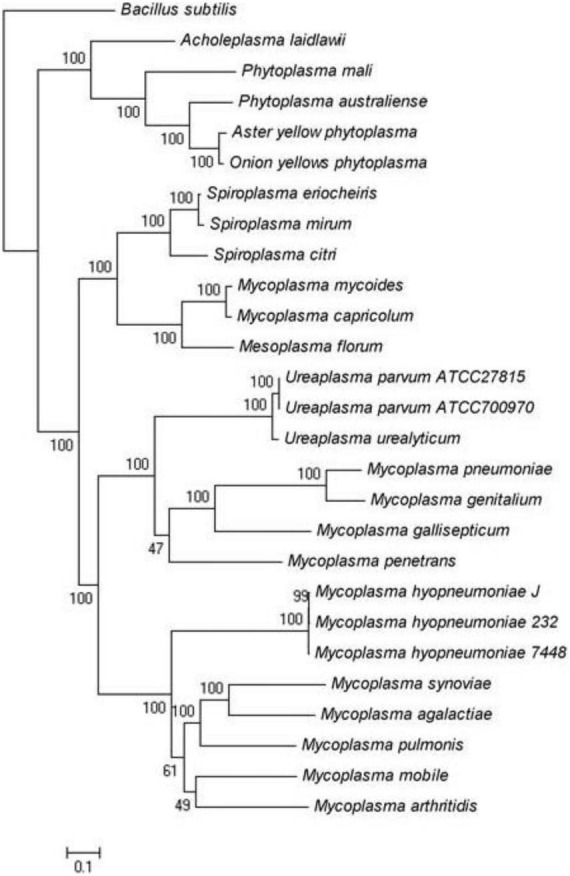
Maximum-likelihood tree of *Spiroplasma* and *Mycoplasma* species for which complete genomes are available.

The genomes of *S. mirum* contain a single, circular chromosome with 1,132,591 bp in length. The base numbering start point was chosen at the first base of the *dnaA* gene, adjacent to the replication origin (*oriC*; Figure 1). The basic characteristics of the genomes of *S. mirum* and *S. eriocheiris* are shown in Table 1. *S. mirum* genome length is 1, 132, 591 bp, and G + C content is 29.41%. *S. mirum* and *S. eriocheiris are* close to each other in the phylogenetic tree (Figure 2), both species could cause cataracts in rats (Hou et al., 2017). The *S. eriocheiris* genome size is 1, 364,757 bp, with 29.79% G + C content (Liu P. et al., 2017). The specific genes of *S. mirum* genomes are listed in Supplementary Table 1. Generally, there is a complete base excision repair system in the *Spiroplasma* genomes. *S. eriocheiris* genome contains several genes involved in homologous recombination, including *recA*, *ruvA/B*, *recD*, *recO*, *recR*, and *recU*, while in *S. mirum* only the *recA* and *recD* genes remain complete (Supplementary Table 2), giving us a hint that *S. mirum* lost the capacity for recombinational repair. We identified 22 transcription factors and one sigma factor in the *S. eriocheiris* genome, and 13 transcription factors and one sigma factor in the *S. mirum* genome (Supplementary Table 3).

**TABLE 1:** General features of *Spiroplasma eriocheiris* and *Spiroplasma mirum* genome.

*Spiroplasma*	*Spiroplasma eriocheiris*	*Spiroplasma mirum*
Genome size (base pairs)	1,364,757 bp	1,132,591 bp
G + C content (%)	29.79	29.41
**Protein coding genes**		
With assigned functions	755	731
Conserved hypothetical	174	138
Hypothetical	313	338
Total	1,242	1,207
Average gene length	976 bp	745 bp
Coding density (%)	88.7%	79.2%
Pseudogenes	53	381
rRNA operons	1	1
tRNA genes	32	33
IS elements	0	3

**FIGURE 3:**
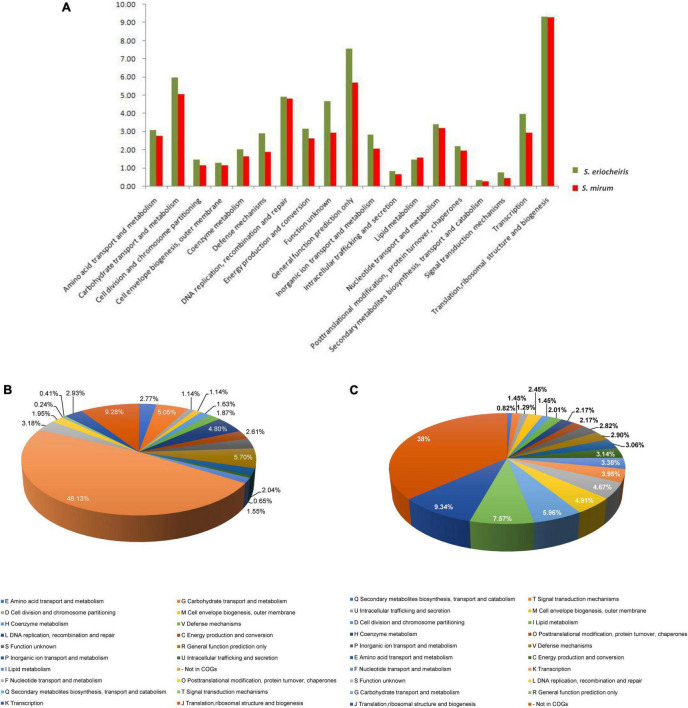
**(A)** Comparison of COG distribution of *Spiroplasma eriocheiris* and *Spiroplasma mirum*. **(B,C)** COG classification of *S. mirum* and *S. eriocheiris*, respectively.

The *S. mirum* contains 1, 207 CDSs, 1 ribosomal RNA operon, and 33 tRNA genes. The average length of CDSs is 745 bp, and CDSs and the totality of rRNA and tRNA genes accounted for 79.2 and 0.6% of the genome, respectively. The GC contents on the GC and the third codon were 30.58 and 20.93%, respectively. A total of 731 CDSs (60.6%) have clear biological functions, 138 CDSs (11.4%) are similar to conservative proteins with unknown function, 338 CDSs (28%) are hypothetical proteins, COG classification was possible for 735 CDSs (60.9%). We assume that the *S. eriocheiris* contains 1, 242 CDSs, with an average length of 976 bp, and the GC contents on the GC and the third codon were 30.66 and 20.65%, respectively. A total of 755 CDSs (60.8%) have been identified, which have clear biological functions, another 174 CDSs (14%) are similar to conservative proteins with unknown function, 313 CDSs (25.2%) are hypothetical proteins, and 769 CDSs (61.9%) can be classified by COG (Figure 3). The genome contains 1 ribosomal RNA operon and 32 tRNA genes. CDSs and stable RNA genes accounted for 88.7 and 0.5% of the genome, respectively. There are no insertion sequences (IS element) or phages in the *S. eriocheiris* genome, while there are three IS elements in the *S. mirum* genome, one of which may be a phage. The phage with a length of 16,008 bp includes 21 genes (From SMM_0576 to SMM_0597). Particularly, SMM_578, SMM_579, SMM_580, SMM_581, and SMM_583 were identified as putative adhesin p58, P12, P54, P123, and P18 of *S. citri*, respectively. Thus, the phage may be acquired from *S. citri.* The phage may have facilitated extensive genome rearrangements in *S. mirum* and contributed to horizontal gene transfers that led to species-specific adaptation to different eukaryotic hosts and acquisition of pathogenicity. In addition, the common ancestor of the *Spiroplasma*, *Entomoplasma*, and *Mycoplasma* clade may have had a relatively large genome and flexible metabolic capacity; the extremely reduced genomes of *Mycoplasma* and *Spiroplasma* species are likely to be the result of independent gene losses (Lo et al., 2013). It is thought that genome reduction is aided by genetic isolation-bacteria that live in monocultures in special host organs, or inside host cells, have less access to other bacterial species from which they can obtain genes (Waterworth et al., 2020).

The genome of *S. mirum* is 232,166 bp smaller than *S. eriocheiris.* Genome reduction is a common phenomenon in numerous pathogens. The pattern of genome reduction observed in *S. atrichopogonis* has been found in several other arthropod-associated bacteria, such as *S. poulsonii* (Lo et al., 2015). Generally, the stable establishment of a bacterium in a host cell results in genome reduction (Campbell et al., 2015). The loss of functions required for living independently but not within a host gives rise to diminished genomes in various symbionts. Although the phenomenon of genome reduction can be explained by existing evolutionary models, the initiation of the process is not thoroughly understood. The key step preceding genome reduction in the symbiont was likely the horizontal acquisition of the putative lagriamide lga biosynthetic gene cluster. The parasitic bacterium does need to produce nutrients that the host provides, leading to the loss of genes that it would need to live independently and to a consequent reduction in genome size (McCutcheon and Moran, 2011).

### Basic metabolic

**FIGURE 4:**
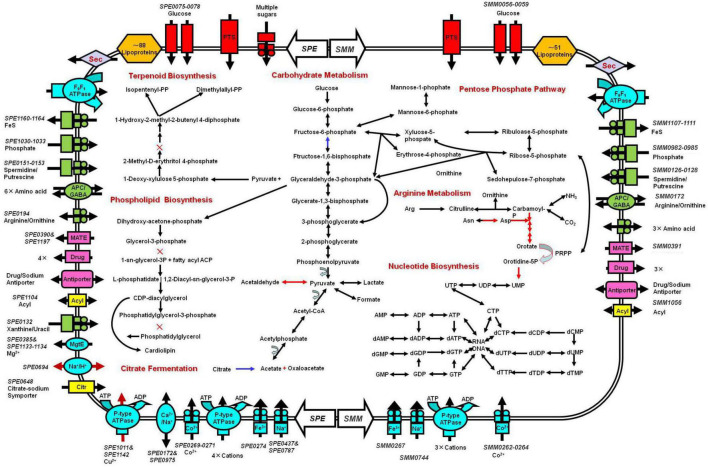
Schematic representation of metabolic pathways of *Spiroplasma eriocheiris* **(left side)** and *Spiroplasma mirum* **(right side)**. Shown are transporters and the main elements of metabolic pathways, deduced from the set of genes with predicted functions. Black arrows: pathways found in both *S. eriocheiris* and *S. mirum* genomes. Blue arrows: *S. eriocheiris* special in *Mollicutes*. Red arrows: pathways found in the *S. eriocheiris* genome but not in the *S. mirum* genome. × : absent enzyme in pathways. Transporters are colored according to their substrates: blue, cations; green, anions and amino acids; rad, carbohydrates; pink, multidrug, and metabolic product efflux. Arrows indicate the direction of substrate transport. In some cases, we cannot identify all the components of these transporters.

Basic feature of *S. mirum* metabolism elucidated by genomic analysis permitted the metabolic reconstruction of spiroplasma (Figure 4). Spiroplasmas are parasitic in specific hosts and their specific tissues, indicating that they have special nutritional requirements and parasitic history. Genomic sequences will provide evidence to reveal the complex nutritional requirement of spiroplasma. Both *S. eriocheiris* and *S. mirum* can utilize glucose in the Embden–Meyerhof–Parnas (EMP) pathway, The oxidative branch of the pentose phosphate pathway was completely absent, as well as the TCA cycle, and finally, pyruvate can be converted into lactic acid, formic acid, and acetic acid. *S. eriocheiris* can also produce acetaldehyde. However, the arginine dihydrolase pathway was found in both strains. Compared with other flexor-membranous microorganisms, *S. eriocheiris* has fructose 1,6-diphosphate fructose in the glycolysis pathway, which can catalyze 1,6-diphosphate to form fructose 6-phosphate in gluconeogenesis. However, due to the absence of citric acid cycle, the gluconeogenesis pathway is still incomplete (Figure 4). Both genomes show a complete absence of pentose phosphate pathway oxidation pathways and incomplete non-oxidation pathways, this is similar to other mycoplasmas (Pollack et al., 2002; Westberg et al., 2004).

The tricarboxylic acid cycle is completely absent in the genome, so do mycoplasma. Arginine hydrolysis pathway exists in both spiroplasma genomes, resulting in ornithine and ATP, CO_2_, and ammonia. Both purine and pyrimidine cannot be synthesized in *S. mirum*, but *S. eriocheiris* can synthesize pyrimidine through 1-diphosphate-5-phosphate ribose (PRPP). The pyrimidine is synthesized. Most amino acids cannot be synthesized in spiroplasma, oxaloacetic acid (produced by citric acid fermentation) can produce aspartic acid, pyruvate can produce serine, asparagine, and glycine can be converted from aspartic acid and serine, respectively. Consistent with their parasitic life characteristics, spiroplasma loses most of their synthetic abilities, such as fatty acids and various cofactors (except CoA). GDSL family lipases can catalyze the decomposition of lipids to obtain fatty acids in *S. eriocheiris* and *S. mirum*, and then fatty acids can be oxidized, but the enzymes that catalyze fatty acids β-oxidation are missing in the genome. terpenoids and phospholipids cannot be synthesized in spiroplasma because of the lack of some enzymes, which must be obtained from the host or medium to maintain their survival (Figure 4).

### Antigen proteins

We used the IgG and antibodies of different antigens in serum can specifically bind to other antigens and Protein A, we use polyclonal antibodies to screen antigens in spiroplasma. In the experiment, *S. mirum* were tested, and negative serum control group was set up to remove non-specific immune binding. We identified 49 and 11 proteins of positive and negative serum, including 9 common proteins. Therefore, 40 antigenic proteins of *S. mirum* were screened, and the results are shown in Table 2. While 45 antigenic proteins of *S. eriocheiris* were identified (Liu Y. et al., 2017). There are six antigenic proteins of *S. mirum* are homologous proteins that can be used as antigen proteins of *S. eriocheiris*, including extension factor EF-G, pyruvate dehydrogenase (β subunit, E2 subunit, E3 subunit), thioredoxin reductase and DegV family proteins.

**TABLE 2:** Immunogenic proteins of *Spiroplasma mirum* identified by anti-*S. mirum* serum and negative serum.

SMM-positive	Gene annotation	SMM-negative
SMM0024	Hypothetical protein	
SMM0033	Single-strand DNA-binding protein	
SMM0064	Translation elongation factor G	
SMM0065	Translation elongation factor Tu	
SMM0066	LemA family protein	SMM0066
SMM0099	F_0_F_1_ ATP synthase subunit epsilon	
SMM0101	Putative lipoprotein	SMM0101
SMM0146	Oligoendopeptidase F	
SMM0156	Heat-inducible transcription repressor HrcA	SMM0156
SMM0159	Molecular chaperone DnaJ	
SMM0170	Arginine deiminase	
SMM0171	Ornithine carbamoyltransferase	SMM0171
SMM0172	Arginine-ornithine antiporter	SMM0172
SMM0233	Adenylosuccinate lyase	
SMM0239	Hypothetical protein	
SMM0299	50S ribosomal protein L17	
SMM0315	Asparagine-tRNA ligase	
SMM0324	Putative lipoate-protein ligase A	SMM0324
SMM0326	Pyruvate dehydrogenase E1 component beta subunit	
SMM0327	Pyruvate dehydrogenase E2 (dihydrolipoamide acetyltransferase) component	
SMM0328	Pyruvate dehydrogenase E3 (dihydrolipoamide dehydrogenase) component	
SMM0388	ABC-type transport system ATP-binding protein	
SMM0422	Endoribonuclease L-PSP	
SMM0437	Hypothetical protein	
	Hypothetical protein	SMM0444
SMM0491	RelA/SpoT family protein	
SMM0532	Truncated transmembrane protein	
SMM0535	Hypothetical protein	
SMM0564	Hypothetical protein	
SMM0585	Hypothetical protein	
SMM0598	hypothetical protein	
SMM0640	Conserved hypothetical protein	
SMM0643	Hypothetical protein	
SMM0693	6-phosphofructokinase	
SMM0696	30S ribosomal protein S2	SMM0696
SMM0834	N-terminal truncated NADH-dependent flavin oxidoreductase	SMM0834
SMM0915	Truncated aldose 1-epimerase	
SMM0928	N-terminal truncated F0F1 ATP synthase subunit C	
SMM0940	Putative phosphate acetyltransferase	
SMM0941	Putative copper homeostasis protein CutC	
SMM1003	Hypothetical protein	
SMM1014	Conserved hypothetical protein	
SMM1020	Putative thioredoxin reductase	
SMM1051	Putative tRNA-binding domain-containing protein	
SMM1054	Putative DegV family protein	
SMM1077	Hypothetical protein	
SMM1121	Lactose phosphotransferase system repressor	
SMM1158	Hypothetical protein	
SMM1170	Fructose-bisphosphate aldolase	
	Cell shape determining protein MreB5	SMM1188
SMM1189	Putative deoxyribonuclease	SMM1189

The oligoendopeptidase F (SMM0146), belonging to thimet oligopeptidase family member, was proved as a surface-exposed proteases of *M. hyopneumoniae* (Jarocki et al., 2019). EF-Tu (SMM_0065) and EF-G (SMM_0064) were identified as antigenic proteins. EF-Tu has evolved the ability to perform diverse functions on the extracellular surface of a wide variety of pathogenic bacteria. While moonlighting functions vary among microbial species, there is a common theme for roles in adherence and in immune regulation (Harvey et al., 2019). EF-G, which is the third most conserved trGTPase among all domains of life. The elongation phase of translation is an important regulatory node in health and disease (Xu et al., 2021). F0F1 ATP synthase (SMM_0099) has transmembrane proton transport function (Feniouk and Junge, 2005) and can be considered candidate antigens to minimize cross reaction in the diagnosis of brucellosis and useful sources for Brucella vaccine development (Ko et al., 2012). Only one lipoprotein (SMM_0101) was screened, lipoprotein is a surface-exposed molecule that is immunodominant and is used as the major antigen for serological diagnosis of *Mycoplasma penetrans* infection (Sasaki et al., 2002). RelA/SpoT family protein (SMM_0491) is considered as enzymes, which control bacterial physiology through synthesis and degradation of the nucleotide alarmone, and acts as toxins of toxin–antitoxin modules (Kurata et al., 2021). Furthermore, putative copper homeostasis protein CutC (SMM_0941) was found in the antigenic protein. Numerous exciting studies have revealed that copper plays an indispensable role in the microbial pathogen-host axis for entities ranging from pathogenic bacteria to deadly fungal species (Li et al., 2019). Fructose 1,6-bisphosphate aldolase is a ubiquitous cytosolic enzyme that catalyzes the fourth step of glycolysis. Apart from their conserved role in carbohydrate metabolism, aldolases have been reported to perform numerous non-enzymatic functions. Fructose-1,6-bisphosphate aldolase of *M. bovis* is a plasminogen-binding adhesion (Gao et al., 2018).

## Discussion

### Comparative genome analysis

We analyzed the genome of *S. mirum*, 2 spiroplasmas, 14 mycoplasmas, 4 phytoplasmas, 3 ureaplasmas, and 1 acholeplasma, which revealed that *S. mirum* share a total of 84 common proteins with them (Supplementary Table 4). *Bacillus subtilis* as an external group. *M. mycoides*, Mycoplasma *capricolum*, and *Mesoplasma florum* have a closer evolutionary relationship to spiroplasma than other *Mollicutes*, this is consistent with the analysis results of the phylogenetic tree constructed with 16S rRNA (Figure 2) (Stülke et al., 2009). We found 684 spiroplasma-specific proteins, including 187 *S. eriocheiris-s*pecific proteins, 182 *S. mirum*-specific proteins, and 315 common proteins exist in two spiroplasma (Supplementary Table 5). No restriction-modification (RM) system was revealed in *Spiroplasma*; thus, the barrier of horizontal gene transfer does not exist. Although the functions of most common proteins are unknown, several adhesion-like lipoprotein may be associated with spiroplasma-specific virulence or host-specific invasion.

**FIGURE 5:**
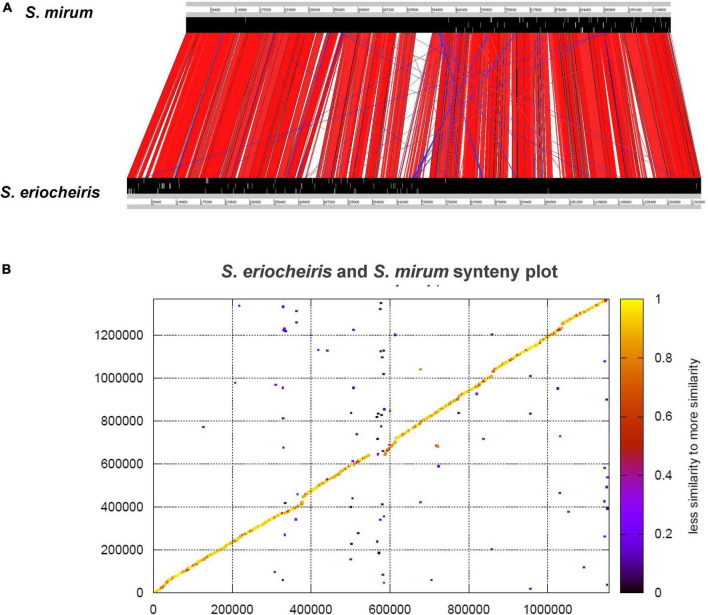
**(A)** Comparison of the gene order between *Spiroplasma eriocheiris* and *Spiroplasma mirum*. **(B)** Protein synteny plot of *S. eriocheiris* and *S. mirum*.

From the comparative analysis of the *S. mirum* and *S. eriocheiris* genomes, it can be found that *S. eriocheiris* genome is 232 kb bigger than *S. mirum*, but those two genomes have a highly conserved genome structure and gene sequence (Figure 5). 273 CDSs of *S. eriocheiris* genomes did not find homologous sequences in *S. mirum*. These CDSs include two lactose PTS systems and some gene clusters. One of the gene clusters functions as citric acid fermentation, consisting of genes such as CitC, CitDEF, CitG, CitS, and CitX, the sodium citrate transporter (CitS) is responsible for the upregulation of citric acid in anaerobic conditions (van der Rest et al., 1992). Citric acid lyase (CitDEF) catalyzes the decomposition of citric acid into acetic acid and oxaloacetic acid. However, oxaloacetic acid cannot further become pyruvate, malic acid, or isocitrate, so citric acid cannot be used as a carbon source and energy source. It is worth to notice that this gene cluster has not been found in the other *Mollicutes*, which means that *S. eriocheiris* may have some special characteristics. Glucuronic acid can be synthesized in *S. eriocheiris*, but it cannot be synthesized in *S. mirum* because of the lack of D-mannose redox enzyme. Another gene cluster catalyzes the first four steps of uracil nucleoside synthesis, from carbamoyl phosphate to lactate 5-phosphate, which is also present in the *M. penetrans* (hf-2) genome (Sasaki et al., 2002). A total of 129 CDSs of *S. mirum* did not find homologous sequences in *S. eriocheiris* genome, and the function of these specific proteins is still unknown.

### Phosphotransferase system

Phosphotransferase system (PTS system) in bacteria consists of two parts: Histidine-containing protein (HPr) that can phosphorylate all carbohydrates and enzyme I. Enzyme II can only catalyze specific substrates, which are composed of 3 functional subunits AII, IIB, IIC, and IID only exist in mannose. Therefore, enzyme II can be divided into four categories: glucose, lactose, mannitol, and mannose. There are 9 categories of PTS systems in the genome of *S. eriocheiris*, including 3 lactose PTS system and 3 fructose PTS system. While in the *S. mirum* genome, most of the genes of the PTS system have been lacking, and only 9 of the 23 genes may have functions except for one glucose and one phage fructose PTS system (Supplementary Table 6). Both fructose and glucose PTS systems are encoded by single genes in both genomes.

### ATP-binding cassette transport system

Because spiroplasma lacks many metabolic and substance synthesis pathways, most of the nutrients they need are obtained from host or artificial medium. The need for a lot of exotic nutrients means that spiroplasma must have a lot of transfer systems. But from the genome, we find that the number of genes in the ATP-binding cassette transport system (ABC transport system) of these two spiroplasma and their proportion to the total number of genes is much less than those of other bacteria, and many are broken genes or pseudogenes. ABC transporters in the genomes of *S. eriocheiris* and *S. mirum* are shown in Supplementary Table 7. Five proteins in *S. eriocheiris* (SPE_0075, SPE_1062, SPE_1069, SPE_1074, and SPE_1075) and one protein in *S. mirum* (SMM_0056) are similar to the solute-binding protein of an ABC transporter in *S. citri*. It has been reported that the ability of *S. citri* to be transmitted by *Circulifer haematoceps* is clearly lost by disruption of a gene encoding a putative solute-binding protein of an ABC transporter and restored by the addition of this gene (Boutareaud et al., 2004). The ATP copper ion transporter in *S. eriocheiris* may be related to the specific infection of mitten crab, because the concentration of copper ion in hemocytes of Chinese mitten crab is particularly high. Moreover *S. eriocheiris* there are more sodium ion-related transporters than *S. mirum*, which is consistent with *S. eriocheiris* physiological characteristics such as high salinity tolerance, because Chinese mitten crab lives in seawater (high salinity) during spawning period.

### Secretion system

The secretory system plays a very important role in the excretion of toxins and pathogenicity in bacteria. The spiroplasma genome only has Sec secretory pathway, which consists of SecA, SecE, SecDF, SecG, SecY, and YidC (Supplementary Table 8). These proteins also constitute the simplest Sec secretion pathway in spiroplasma, although the Sec secretion pathway of spiroplasma is incomplete when compared to the secretion pathway in *Escherichia coli*, these proteins are consistent with the minimal required set of secretion and translocation proteins. The genome analysis indicates that the composition of Sec secretion pathway of spiroplasma is very similar to that of mycoplasma, but the important feature is the lack of SecB proteins in spiroplasma. It is speculated that there are other molecular chaperones to perform SecB functions, like *Bacillus subtilis*. The other special thing is that most of the *Mollicutes* have double-functional SecDF proteins. It is located on the membrane and pumps secretory proteins out of the membrane by hydrolysis ATP with SecA and SecY support. Because the spiroplasma have very special motility, the contribution of Sec system in spiroplasma motility is not as great as mycoplasma.

Signal peptidase is divided into signal peptidase I and signal peptidase II, which are responsible for disconnecting signal peptides at the N terminal of protein, but we only found signal peptidase II in the spiroplasma genome, the same as other *Mollicutes* members. Signal peptidase is considered to play an important role in the pathogenesis of *Mollicutes*, because it plays a crucial role in the formation of adhesion proteins in *M. hyopneumoniae* and *M. pneumoniae*; 37 and 15 extracellular proteins were found in *S. eriocheiris* and *S. mirum*, respectively. These proteins may be secreted by the Sec system, but the function of most proteins is unknown. Besides the hypothetical proteins and transporters, endonuclease I and GDSL family lipases have been found in *S. eriocheiris* genome and these two proteins may play a role in host invading. Verotoxin, a known functional extramembrane protein, which is an important toxin in bacteria, was found in *S. mirum*.

### Outer membrane proteins and virulence proteins

There are the *S. mirum* contains 243 membrane proteins, 51 lipoproteins, and 15 extra-membrane proteins, while 282 membrane proteins, 88 lipoproteins, and 37 extra-membrane proteins in the genome of *S. eriocheiris*. Lipoproteins are usually located on the surface of the cell membrane of Gram-positive bacteria, but because spiroplasma evolved from clostridium, and has no cell wall. Lipoproteins located in the outer membrane of spiroplasma are considered to be involved in the colonization of the hosts, ensuring vertical transmission (Paredes et al., 2015). While only one lipoprotein (SMM0101) was identified by antigen proteomics. This result may imply that not all of the lipoprotein is immunogenic. Spiralin is the most studied protein in spiroplasma, and it is also a unique protein in spiroplasma. There are two Spiralin protein (SPE0615 and SPE1172) in the genome of *S. eriocheiris*, while only 87 bp fragments at the C terminal of one Spiralin protein exist in the *S. mirum*. The similarity of Spiralin protein between *S. mirum, S. citri*, *Spiroplasma kunkelii*, *Spiroplasma melliferum* and *Spiroplasma phoenicerum* is low, which may be caused by the difference of host and parasitic environment, and the Spiralin protein has polymorphism.

The Pre-lipoprotein acetyltransferase is a membrane lipoprotein, which can catalyze the first step of lipoprotein synthesis, transfer the acetyl group to the N terminal of the protein, and connect with cysteine to form lipoprotein. Prolipoprotein diacylglyceryl transferase (Lgt) and lipoprotein signal peptidase II (Lsp II) are unique enzymes (Hutchings et al., 2009) and associated with lipoprotein metabolism in prokaryotes. Alterations in the expression of pre-lipoprotein acetyltransferase and signal peptidase can cause changes in virulence, transport, and signaling systems. There are two Lgt and one Lsp II encoding genes in both genomes of *S. eriocheiris* and *S. mirum* (Supplementary Table 9).

According to the genome analysis, there are two kinds of adhesion proteins (SARPs) (SPE0025 and SPE0529) in *S. eriocheiris* (Supplementary Table 9). Adhesion proteins play a very important role in the interaction between spiroplasma and insect cells (Yu et al., 2000). The transposons in *S. mirum* contain five adhesion-like proteins (SMM_0578-0581 and SMM_0583; Supplementary Table 9), these proteins are homologous to P58, P123, P54, and P18 in *S. citri*, and these proteins are specific in spiroplasma (Bai and Hogenhout, 2002). The ability of *S. citri* to infect leafhoppers depends on a protein, identified as a substrate-binding protein for ABC transporters (Boutareaud et al., 2004). There are 5 Substrate-binding proteins (SPE0075, SPE 1062, SPE 1069, SPE 1074, and SPE 1075) and 1 ABC transporters homologous (SMM0056) present in *S. eriocheiris* and *S. mirum*. Those proteins may promote the invasion process.

Endo-beta-N-acetylglucosaminidase (SPE_0847 and SMM_ 0801) was shown to be a pathogenicity determinant of mammal pathogens and depressed immune response and interfered with host defense (Valisena et al., 1991). SPE_0813 and SMM_0767 encoded a truncated toxin protein, which is lethal to mice and toxic to Vero cells (Amimoto et al., 2007). SPE_0973 encodes a truncated toxin A, which is *S. eriocheiris* specific and is homologous with the N-terminal of toxin A of *Clostridium difficile*. SMM_0541 encodes an extracellular verocytotoxin 1, which is *S. mirum* specific and shows homology with the shiga toxin 1A subunit (Supplementary Table 9). The presence of domains similar to proteins of the incomplete sec secretion system in pathogenic bacteria suggests that *S. mirum* possesses a related translocation system. The Virulence substances may be delivered by the sec secretion system.

FeoB, a high-affinity ferrous iron uptake membrane protein, is present in many bacteria except for Mollicute genomes and contributes considerably to bacterial virulence (Cartron et al., 2006). We identified a FeoB-encoding gene in spiroplasma genomes (SPE_0274 and SMM_0267), which might enable spiroplasmas to compete for iron from the host and contribute to their pathogenicity.

Two copper-transporting P-type ATPases (SPE_1011, SPE_1142) of *S. eriocheiris* might be associated with its pathogenicity and host specificity. It is known that *S. eriocheiris* is transmitted in blood of crabs (Wang et al., 2004), which consists of a large amount of copper cation. NADH peroxidase (SMM_0037) and putative thiol peroxidase (SMM_1137 and SMM_0497) were found in the genome of *S. mirum*, these enzymes may protect *S. mirum* from oxidative. The presence of domains similar to proteins of the sec secretion system in pathogenic bacteria suggests that spiroplasma possesses a related translocation system.

### Genetic degradation

*Spiroplasma mirum* have 383 pseudogenes, which covered 11.7% genome length, 31.6% gene numbers (Supplementary Table 10). This indicates that the genome of *S. mirum* is degenerating. In contrast, only 53 pseudogenes were revealed in the *S. eriocheiris* genome, and 23 pseudogenes can be compensated for by other collateral homologous genes. We found 282 genes in the genome of *S. eriocheiris* constitute 90 collateral homologous gene families, while 188 genes in *S. mirum* can form 56 collateral homologous gene families (Supplementary Table 11). The complete collateral homologous genes of 96 pseudogenes in *S. mirum* were found. This means that other pseudogenes are not necessary for *S. mirum*. The main function of pseudogenes is related to carbohydrate transport and metabolism in *S. mirum*. The encoding density of *S. mirum* genome is 79.2%, which is much lower than that of other bacteria. A large amount of non-coding DNA may be caused by genetic degradation. The GC content of the non-coding regions in *S. eriocheiris* and *S. mirum* was 22.9 and 24.9%, respectively, which was lower than the coding region. Pseudogenes may be caused by previously important genes that are not strictly evolved and gradually accumulated mutations (Lawrence et al., 2001). Usually, the high deletion rate of chromosomes will lead to the formation of pseudogenes, and the intracellular bacterium will lead to an increase in deletion rate and mutation rate due to the constant environment. However, the limited function of mismatch repair system and the deletion of homologous recombinant gene may be the main reasons for the increase in pseudogenes. The pathogen has acquired genes from other bacteria despite going through genome reduction, suggesting that isolation has not yet played a major role in this case of genome reduction, with horizontal gene gains still offering a potential route for adaptation (Waterworth et al., 2020).

### Horizontal transfer genes

Because there is no restriction modification system in the spiroplasma genome, the hindrance of horizontal transfer gene does not exist. 51 *S. eriocheiris* proteins and 15 *S. mirum* proteins are homologous to non-mollicutes bacteria (Supplementary Table 12). These genes may obtain from other bacteria genes after the evolution of spiroplasma. These horizontal transfer genes can be divided into 6 gene clusters in *S. eriocheiris* genome, including 3 PTS systems, 1 citric acid fermentation and 1 glucuronic acid fermentation. The acquisition of some horizontal transfer genes represents some special properties of spiroplasma, such as fructose 1,6-bisphosphate enzyme mentioned in metabolism. In addition, we also found some virulence factors in the horizontal transfer system. Endo-Beta-N-acetylglucosidase has been found in both spiroplasma genome, which is an important pathogenic factor of animal pathogen and can reduce host immune function (Valisena et al., 1991). Both *S. eriocheiris* and *S. mirum* contain this protein consistent with their characteristics as animal pathogen.

One disconnected toxin protein was found in *S. eriocheiris* (SPE_0319) and *S. mirum* (SMM_0306) genomes and which is homologous to the N terminal of the cytotoxic TpeL protein of *Clostridium perfringens*. TpeL protein, a novel virulence factor, plays an important role in mouse lethal processes (Amimoto et al., 2007). Because the N terminal sequence is conserved in many clostridium toxins, we speculate that TpeL protein may be an important virulence factor of spiroplasma. A disconnected toxin A protein is also present in the genome of *S. eriocheiris*, which is homologous to the N terminal of *Clostridium difficile* toxin A protein. As discussed in Sec system, the presence of Verotoxin I (SMM_0541) in the genome of *S. mirum*, which is homologous to the virulence factors of *Shigella dysenteriae* and Shigatoxigenic *E. coli*, named shigella toxin IA subunit. Ferritin FeoB is a very important virulence factor in many bacteria. SPE0274 and SMM0267 can encode complete FeoB, which may be helpful for spiroplasma to despoil ions in host and produce virulence. These horizontal transfer genes do not exist in other *Mollicutes* members but are important virulence factors in some *Clostridium* and *bacilli*. This reveals that the pathogenic characteristics of spiroplasma are similar to intestinal pathogens such as *Clostridium* or *bacillus*.

The *S. eriocheiris* and *S. mirum* genomics research reveals the commonness and difference in spiroplasma in aquatic environment and terrestrial environment. In an evolutionary sense, we can continue to study the evolutionary origin and classification of cell wall-less microbes in the *Mollicutes*, as well as their differences and evolutionary relationships with other bacteria. Further genomic analysis of two spiroplasma may identify factors associated with their special living environment (parasitic history) and pathogenicity due to aquatic and terrestrial habitat differences.

### Antigen proteomics

Antigen proteomics is a new research field of pathogen in recent years. At present, there is still less study of spiroplasma antigen proteomics, and the study of mycoplasma antigen proteomics has just begun. The current research on mycoplasma has *M. genitalium*, *M. pneumoniae*, *M. hyopneumoniae*, and *M. synoviae*. *S. mirum* belongs to serological group V, while *S. eriocheiris* belongs to a new *Spiroplasma* group XLIII (Wang et al., 2011). The two species have very different hosts and environments. So, it is not surprising that these two strains have very different cell surface proteins and antigen-related genes. We used immunoprecipitation to screen the antigenic proteins of *S. mirum*. Negative serum was used as a control to determine the main antigen protein. We’ve screened out 40 antigen proteins of *S. mirum* were screened out, six of them are homologous proteins that can be used as antigen proteins of *S. eriocheiris*, including extension factor EF-G, pyruvate dehydrogenase (β subunit, E2 subunit, and E3 subunit), thioredoxin reductase, and DegV family proteins. Elongation factor thermo unstable (EF-Tu) and pyruvate dehydrogenase E1-β subunit are also *M. hyopneumoniae* antigen proteins (Pinto et al., 2007). The EF-Tu is also the antigen protein of *M. synoviae* (Bercic et al., 2008). Mmm SC PG1 of *M. mycoides* shared with extraordinary EF-Tu, extension factors EF-G, pyruvate dehydrogenase, fatty acids protein ligase A and fructose diphosphate aldolase as antigen proteins (Jores et al., 2009), *M. mycoides* subsp. mycoides strain B237 shared pyruvate dehydrogenase as antigen protein (Naseem et al., 2010). In addition, translation elongation factor G is likely to be a drug target for the malarial parasite. Therefore, the EF-G could be evaluated as potential proteins of diagnostic markers and target proteins of research on pathogenicity (Liu Y. et al., 2017).

Previously, we have screened out 45 antigen proteins of *S. eriocheiris* (Liu Y. et al., 2017), many of which are homologous to proteins found in mycoplasma antigen proteomics, such as pyruvate dehydrogenase E1-β subunit is also *M. pneumoniae* antigen protein (Pinto et al., 2007); enolases, ATP synthetases β subunits, pyruvate kinases, and DanK can act as antigen proteins of *M. synoviae* (Bercic et al., 2008). *Mycoplasma*, which has the closest evolutionary relationship with spiroplasma, has been studied in immunoproteomics. Jores et al. screened the immunogenic protein of *M. mycoides* subsp. mycoides small clonal strain MmmSC PG1T and used experiments. The serum prepared in the laboratory screened out 24 immunogenic proteins. 11 of these 24 antigenic proteins were homologous to the antigenic proteins of *S. eriocheiris* sinensis. They are elongation factor-G, leucyl peptidase, Pyruvate dehydrogenase (α subunit, β subunit, E2 subunit, and E3 subunit), acetate kinase, glyceraldehyde 3-phosphate dehydrogenase, triose phosphate isomerase, phosphoglycerate mutase, and ATP synthase β subunit and others (Jores et al., 2009). Naseem et al. screened 22 immunogenic proteins in the small clonal strain B237 of *M. mycoides* subsp. mycoides, including DNA-guided RNA polymerase proteins such as β subunit and acyl carrier protein phosphodiesterase, are also antigen proteins of *S. eriocheiris* (Naseem et al., 2010). The other antigenic proteins specific to the microbes of the class lamina are mostly lipoproteins or membrane proteins with variable morphology and structure. A putative copper homeostasis protein CutC (SMM0941) was identified in the antigenic protein. The bacterial copper efflux system plays a predominant role in regulating pathogen fitness during infection. Analyses of copper homeostasis in bacteria and fungi extensively demonstrate that copper is utilized by the host immune system as an anti-microbial agent. The expression of copper efflux and detoxification from microbial pathogens is induced to counteract the host’s copper bombardment, which in turn disrupts these machineries, resulting in the attenuation of microbial survival in host tissue (Li et al., 2019).

## Conclusion

Now we have entered the post-genome era because there are no technical obstacles to genome sequencing, so proteomics (including immunoproteomics and antigenic histology) has also entered a period of rapid development. Regarding pathogenic microorganisms, people are most concerned about which proteins of microorganisms play a major role in the pathogenic process, so it is of great significance for us to carry out this antigen-screening experiment. However, we only preliminarily screened the antigen proteins of spiroplasma, and the specific functions of each protein need to be further studied.

Spiroplasma was attractive for research due to their very small, helical structure and unusual mode of motility by means of a contractile cytoskeleton-like structure, functioning as a linear motor (Trachtenberg, 2006; Trachtenberg et al., 2008). Some structural, dynamic, and proteomic approaches have elucidated the basic organizing principles of their minimal, yet functional, cytoskeleton-like architecture (Liu P. et al., 2017). The full genome information of spiroplasma should provide further significant opportunities to understand the pathogenesis and mechanism of this unique cytoskeleton-like architecture. The comparative genomic analysis of *S. eriocheiris* and *S. mirum* reveals some remarkable differences in genes related to their special characteristics associated with their living environments. The complete genome sequence of *S. mirum* from rabbit tick reveals that Glycolysis, pentose phosphate pathway, arginine metabolism, nucleotide biosynthesis, and citrate fermentation were found in *S. mirum*. Trichloroacetic acid, fatty acids metabolism, phospholipid biosynthesis, terpenoid biosynthesis, and cofactors synthesis were completely absent. But lactose-specific PTS was not identified in *S. mirum*. A total of 273 *S. eriocheiris* CDSs had no homologous in *S. mirum* genome, including lactose-specific PTS and citrate fermentation gene cluster. *S. mirum* genome had 381 pseudogenes, accounting for 31.6% of total protein-coding genes. This is the evidence that spiroplasma genome is under an ongoing genome reduction. Interestingly, *S. mirum* was infective for suckling mice and produced cataracts. But we could not find genes associated with this character up to now. However, further analyses and experiments based on the genomic data should be helpful to probe the mechanisms of *S. mirum* infections in vertebrate hosts.

We identified the antigen proteins and antigen membrane proteins of *S. mirum*. 40 proteins in *S. mirum* are identified in relation to virulence. The results reported in this study will elucidate the immune relationship between the host and the pathogen *S. mirum*, as well as benefit analytical techniques for identifying bioindicators and more precise diagnosis of the pathology. Currently, there is no explicit understanding of the exact role of these potential immunogenic proteins; future experiments should reveal the immunogenicity of these immunogenic proteins, and the interaction between these proteins and host proteins can be identified and proved.

## Data availability statement

The datasets presented in this study can be found in online repositories. The names of the repository/repositories and accession number(s) can be found in the article/Supplementary material.

## Author contributions

PL, YXL, YY, JC, WS, WG (Antigen proteome analysis parts), RL, QZ, YAL, WW, QM, JO, and ZY (Genome part) contributed to the conception and design. PL, YXL (Antigen proteome analysis parts), and YY (Genome part) contributed to the collection and assembly of data. PL, YXL, and YY wrote the manuscript. PL, WS, and WG approved the final manuscript. All authors contributed to the article and approved the submitted version.

## Footnotes

1
http://www.phrap.org/phredphrapconsed.html

## References

[B1] Amimoto K. Noro T. Oishi E. Shimizu M. (2007). A novel toxin homologous to large clostridial cytotoxins found in culture supernatant of *Clostridium perfringens* type C. *Microbiology* 153(Pt 4), 1198–1206. 10.1099/mic.0.2006/002287-0 17379729

[B2] Aslam B. Basit M. Nisar M. A. Khurshid M. Rasool M. H. (2017). Proteomics: Technologies and their applications. *J. Chromatogr. Sci.* 55 182–196. 28087761 10.1093/chromsci/bmw167

[B3] Bai X. Hogenhout S. A. (2002). A genome sequence survey of the mollicute corn stunt spiroplasma *Spiroplasma kunkelii*. *FEMS Microbiol. Lett.* 210 7–17. 10.1111/j.1574-6968.2002.tb11153.x 12023071

[B4] Bastian F. O. Sanders D. E. Forbes W. A. Hagius S. D. Walker J. V. Henk W. G. (2007). Spiroplasma spp. from transmissible spongiform encephalopathy brains or ticks induce spongiform encephalopathy in ruminants. *J. Med. Microbiol.* 56(Pt 9), 1235–1242. 17761489 10.1099/jmm.0.47159-0

[B5] Bercic R. L. Slavec B. Lavric M. Narat M. Bidovec A. Dovc P. (2008). Identification of major immunogenic proteins of *Mycoplasma synoviae* isolates. *Vet. Microbiol.* 127 147–154. 10.1016/j.vetmic.2007.07.020 17720337

[B6] Boutareaud A. Danet J. L. Garnier M. Saillard C. (2004). Disruption of a gene predicted to encode a solute binding protein of an ABC transporter reduces transmission of *Spiroplasma citri* by the leafhopper *Circulifer haematoceps*. *Appl. Environ. Microbiol.* 70 3960–3967. 10.1128/AEM.70.7.3960-3967.2004 15240270 PMC444794

[B7] Campbell M. A. Van Leuven J. T. Meister R. C. Carey K. M. Simon C. McCutcheon J. P. (2015). Genome expansion via lineage splitting and genome reduction in the cicada endosymbiont Hodgkinia. *Proc. Natl. Acad. Sci. U.S.A.* 112 10192–10199. 10.1073/pnas.1421386112 26286984 PMC4547289

[B8] Cartron M. Maddocks S. Gillingham P. Craven C. Andrews S. (2006). Feo - transport of ferrous iron into bacteria. *Biometals* 19 143–157. 16718600 10.1007/s10534-006-0003-2

[B9] Carver T. J. Rutherford K. M. Berriman M. Rajandream M. A. Barrell B. G. Parkhill J. (2005). ACT: The artemis comparison tool. *Bioinformatics* 21 3422–3423. 15976072 10.1093/bioinformatics/bti553

[B10] Castresana J. (2000). Selection of conserved blocks from multiple alignments for their use in phylogenetic analysis. *Mol. Biol. Evol.* 17 540–552. 10742046 10.1093/oxfordjournals.molbev.a026334

[B11] Chen S. Hao H. Yan X. Liu Y. Chu Y. (2019). Genome-wide analysis of *Mycoplasma dispar* provides insights into putative virulence factors and phylogenetic relationships. *G3* 9 317–325. 10.1534/g3.118.200941 30573467 PMC6385981

[B12] Delcher A. L. Harmon D. Kasif S. White O. Salzberg S. L. (1999). Improved microbial gene identification with GLIMMER. *Nucleic Acids Res.* 27 4636–4641. 10556321 10.1093/nar/27.23.4636 PMC148753

[B13] Edgar R. (2004). MUSCLE: A multiple sequence alignment method with reduced time and space complexity. *BMC Bioinformatics* 5:113. 10.1186/1471-2105-5-113 15318951 PMC517706

[B14] Feniouk B. A. Junge W. (2005). Regulation of the F0F1-ATP synthase: The conformation of subunit epsilon might be determined by directionality of subunit gamma rotation. *FEBS Lett.* 579 5114–5118. 10.1016/j.febslet.2005.08.030 16154570

[B15] Gao X. Bao S. Xing X. Fu X. Zhang Y. Xue H. (2018). Fructose-1,6-bisphosphate aldolase of *Mycoplasma bovis* is a plasminogen-binding adhesin. *Microb. Pathog.* 124 230–237. 10.1016/j.micpath.2018.08.032 30142464

[B16] Guindon S. Gascuel O. (2003). A simple, fast, and accurate algorithm to estimate large phylogenies by maximum likelihood. *Syst. Biol.* 52 696–704. 14530136 10.1080/10635150390235520

[B17] Guo F. B. Ou H. Y. Zhang C. T. (2003). ZCURVE: A new system for recognizing protein-coding genes in bacterial and archaeal genomes. *Nucleic Acids Res.* 31 1780–1789. 10.1093/nar/gkg254 12626720 PMC152858

[B18] Harne S. Duret S. Pande V. Bapat M. Béven L. Gayathri P. (2020). MreB5 is a determinant of rod-to-helical transition in the cell-wall-less bacterium spiroplasma. *Curr. Biol.* 30 4753–4762.e7. 10.1016/j.cub.2020.08.093 32976813

[B19] Harvey K. L. Jarocki V. M. I Charles G. Djordjevic S. P. (2019). The diverse functional roles of elongation factor Tu (EF-Tu) in microbial pathogenesis. *Front. Microbiol.* 10:2351. 10.3389/fmicb.2019.02351 31708880 PMC6822514

[B20] Hou L. Gu W. Zhu H. Yao W. Wang W. Meng Q. (2017). *Spiroplasma eriocheiris* induces mouse 3T6-Swiss albino cell apoptosis that associated with the infection mechanism. *Mol. Immunol.* 91 75–85. 10.1016/j.molimm.2017.08.002 28889064

[B21] Hutchings M. I. Palmer T. Harrington D. J. Sutcliffe I. C. (2009). Lipoprotein biogenesis in Gram-positive bacteria: Knowing when to hold ‘em, knowing when to fold ‘em. *Trends Microbiol.* 17 13–21. 10.1016/j.tim.2008.10.001 19059780

[B22] Itoh K. I Pan J. Koshimizu K. (1989). A proposed life cycle model of *Spiroplasma mirum* based on scanning electron microscopical observations of growth in liquid culture. *Microbiol. Immunol.* 33 821–832. 10.1111/j.1348-0421.1989.tb00968.x 2615674

[B23] Jarocki V. M. Raymond B. B. A. Tacchi J. L. Padula M. P. Djordjevic S. P. (2019). *Mycoplasma hyopneumoniae* surface-associated proteases cleave bradykinin, substance P, neurokinin A and neuropeptide Y. *Sci. Rep.* 9:14585. 10.1038/s41598-019-51116-w 31601981 PMC6787215

[B24] Jores J. Meens J. Buettner F. F. Linz B. Naessens J. Gerlach G. F. (2009). Analysis of the immunoproteome of *Mycoplasma mycoides* subsp. *mycoides* small colony type reveals immunogenic homologues to other known virulence traits in related *Mycoplasma* species. *Vet. Immunol. Immunopathol.* 131 238–245. 10.1016/j.vetimm.2009.04.016 19443045

[B25] Juncker A. S. Willenbrock H. Von Heijne G. Brunak S. Nielsen H. Krogh A. (2003). Prediction of lipoprotein signal peptides in Gram-negative bacteria. *Protein Sci.* 12 1652–1662. 12876315 10.1110/ps.0303703 PMC2323952

[B26] Kanehisa M. Goto S. Kawashima S. Okuno Y. Hattori M. (2004). The KEGG resource for deciphering the genome. *Nucleic Acids Res.* 32 D277–D280. 14681412 10.1093/nar/gkh063 PMC308797

[B27] Khan F. A. Zhao G. Guo Y. Faisal M. Chao J. Chen X. (2018). Proteomics identification and characterization of MbovP730 as a potential DIVA antigen of *Mycoplasma bovis*. *Oncotarget* 9 28322–28336. 10.18632/oncotarget.22265 29983863 PMC6033335

[B28] Ko K. Y. Kim J. W. Her M. Kang S. I. Jung S. C. Cho D. H. (2012). Immunogenic proteins of *Brucella abortus* to minimize cross reactions in brucellosis diagnosis. *Vet. Microbiol.* 156 374–380. 10.1016/j.vetmic.2011.11.011 22192360

[B29] Kurata T. Brodiazhenko T. Alves Oliveira S. R. Roghanian M. Sakaguchi Y. Turnbull K. J. (2021). RelA-SpoT Homolog toxins pyrophosphorylate the CCA end of tRNA to inhibit protein synthesis. *Mol. Cell* 81 3160–3170.e9. 10.1016/j.molcel.2021.06.005 34174184

[B30] Kürner J. Frangakis A. S. Baumeister W. (2005). Cryo-electron tomography reveals the cytoskeletal structure of *Spiroplasma melliferum*. *Science* 307 436–438. 10.1126/science.1104031 15662018

[B31] Lawrence J. G. Hendrix R. W. Casjens S. (2001). Where are the pseudogenes in bacterial genomes? *Trends Microbiol.* 9 535–540. 11825713 10.1016/s0966-842x(01)02198-9

[B32] Li C. Li Y. Ding C. (2019). The role of copper homeostasis at the host-pathogen axis: From bacteria to fungi. *Int. J. Mol. Sci.* 20:175. 10.3390/ijms20010175 PMC6337107 30621285

[B33] Liu P. Du J. Zhang J. Wang J. Gu W. Wang W. (2018). The structural and proteomic analysis of *Spiroplasma eriocheiris* in response to colchicine. *Sci. Rep.* 8:8577. 10.1038/s41598-018-26614-y 29872058 PMC5988712

[B34] Liu P. Hou L. Liu M. Xu X. Gao Q. Deng J. (2020). Phosphoproteomic analysis of *Spiroplasma eriocheiris* and crosstalk with acetylome reveals the role of post-translational modifications in metabolism. *Curr. Proteomics* 17 392–403.

[B35] Liu P. Zheng H. Meng Q. Terahara N. Gu W. Wang S. (2017). Chemotaxis without conventional two-component system, based on cell polarity and aerobic conditions in helicity-switching swimming of *Spiroplasma eriocheiris*. *Front. Microbiol.* 8:58. 10.3389/fmicb.2017.00058 28217108 PMC5289999

[B36] Liu Y. Xu Y. Li S. Xu X. Gao Q. Yuan M. (2017). Identification of proteome, antigen protein and antigen membrane protein from *Spiroplasma eriocheiris*. *Lett. Appl. Microbiol.* 65 395–402. 10.1111/lam.12784 28763106

[B37] Lo W. S. Chen L. L. Chung W. C. Gasparich G. E. Kuo C. H. (2013). Comparative genome analysis of *Spiroplasma melliferum* IPMB4A, a honeybee-associated bacterium. *BMC Genomics* 14:22. 10.1186/1471-2164-14-22 23324436 PMC3563533

[B38] Lo W. S. Gasparich G. E. Kuo C. H. (2015). Found and lost: The fates of horizontally acquired genes in arthropod-symbiotic spiroplasma. *Genome Biol. Evol.* 7 2458–2472. 10.1093/gbe/evv160 26254485 PMC4607517

[B39] Lowe T. M. Eddy S. R. (1997). tRNAscan-SE: A program for improved detection of transfer RNA genes in genomic sequence. *Nucleic Acids Res.* 25 955–964. 9023104 10.1093/nar/25.5.955 PMC146525

[B40] McCutcheon J. P. Moran N. A. (2011). Extreme genome reduction in symbiotic bacteria. *Nat. Rev. Microbiol.* 10 13–26. 22064560 10.1038/nrmicro2670

[B41] Megraud F. Gamon L. B. McGarrity G. J. (1983). Characterization of *Spiroplasma mirum* (suckling mouse cataract agent) in a rabbit lens cell culture. *Infect. Immun.* 42 1168–1175. 10.1128/iai.42.3.1168-1175.1983 6642663 PMC264421

[B42] Naseem S. Meens J. Jores J. Heller M. Dübel S. Hust M. (2010). Phage display-based identification and potential diagnostic application of novel antigens from *Mycoplasma mycoides* subsp. *mycoides* small colony type. *Vet. Microbiol.* 142 285–292. 10.1016/j.vetmic.2009.09.071 19900769

[B43] Nunan L. M. Lightner D. V. Oduori M. A. Gasparich G. E. (2005). *Spiroplasma penaei* sp. nov., associated with mortalities in *Penaeus vannamei*, Pacific white shrimp. *Int. J. Syst. Evol. Microbiol.* 55(Pt 6), 2317–2322. 10.1099/ijs.0.63555-0 16280489

[B44] Paredes J. C. Herren J. K. Schüpfer F. Marin R. Claverol S. Kuo C. H. (2015). Genome sequence of the *Drosophila melanogaster* male-killing *Spiroplasma* strain MSRO endosymbiont. *mBio* 6:e02437-14. 10.1128/mBio.02437-14 25827421 PMC4453565

[B45] Pinto P. M. Chemale G. de Castro L. A. Costa A. P. Kich J. D. Vainstein M. H. (2007). Proteomic survey of the pathogenic *Mycoplasma hyopneumoniae* strain 7448 and identification of novel post-translationally modified and antigenic proteins. *Vet. Microbiol.* 121 83–93. 10.1016/j.vetmic.2006.11.018 17182197

[B46] Pollack J. D. Myers M. A. Dandekar T. Herrmann R. (2002). Suspected utility of enzymes with multiple activities in the small genome *Mycoplasma* species: The replacement of the missing household nucleoside diphosphate kinase gene and activity by glycolytic kinases. *Omics* 6 247–258. 10.1089/15362310260256909 12427276

[B47] Rasko D. A. Myers G. S. Ravel J. (2005). Visualization of comparative genomic analyses by BLAST score ratio. *BMC Bioinformatics* 6:2. 10.1186/1471-2105-6-2 15634352 PMC545078

[B48] Sasajima Y. Miyata M. (2021). Prospects for the mechanism of spiroplasma swimming. *Front. Microbiol.* 12:706426. 10.3389/fmicb.2021.706426 34512583 PMC8432965

[B49] Sasaki Y. Ishikawa J. Yamashita A. Oshima K. Kenri T. Furuya K. (2002). The complete genomic sequence of *Mycoplasma penetrans*, an intracellular bacterial pathogen in humans. *Nucleic Acids Res.* 30 5293–5300. 10.1093/nar/gkf667 12466555 PMC137978

[B50] Shaevitz J. Lee J. Fletcher D. (2005). *Spiroplasma* swim by a processive change in body helicity. *Cell* 122 941–945. 10.1016/j.cell.2005.07.004 16179261

[B51] Stülke J. Eilers H. Schmidl S. R. (2009). “Mycoplasma and spiroplasma,” in *Encyclopedia of microbiology*, 3rd Edn, ed. Schaechter M. (Oxford: Elsevier), 208–219.

[B52] Sussman H. E. (2015). 20 years of genome research. Preface. *Genome Res.* 25:xv. 10.1101/gr.199026.115 26430164 PMC4579344

[B53] Takahashi D. Fujiwara I. Miyata M. (2020). Phylogenetic origin and sequence features of MreB from the wall-less swimming bacteria Spiroplasma. *Biochem. Biophys. Res. Commun.* 533 638–644. 10.1016/j.bbrc.2020.09.060 33066960

[B54] Trachtenberg S. (2004). Shaping and moving a spiroplasma. *J. Mol. Microbiol. Biotechnol.* 7 78–87. 15170406 10.1159/000077872

[B55] Trachtenberg S. (2006). The cytoskeleton of spiroplasma: A complex linear motor. *J. Mol. Microbiol. Biotechnol.* 11 265–283. 10.1159/000094060 16983201

[B56] Trachtenberg S. Dorward L. M. Speransky V. V. Jaffe H. Andrews S. B. Leapman R. D. (2008). Structure of the cytoskeleton of *Spiroplasma melliferum* BC3 and its interactions with the cell membrane. *J. Mol. Biol.* 378 778–789. 10.1016/j.jmb.2008.02.020 18400234

[B57] Uchiyama I. (2003). MBGD: Microbial genome database for comparative analysis. *Nucleic Acids Res.* 31 58–62. 12519947 10.1093/nar/gkg109 PMC165556

[B58] Valisena S. Varaldo P. Satta G. (1991). Staphylococcal endo-beta-N-acetylglucosaminidase inhibits response of human lymphocytes to mitogens and interferes with production of antibodies in mice. *J. Clin. Invest.* 87 1969–1976. 10.1172/JCI115224 1904069 PMC296950

[B59] van der Rest M. E. Siewe R. M. Abee T. Schwarz E. Oesterhelt D. Konings W. N. (1992). Nucleotide sequence and functional properties of a sodium-dependent citrate transport system from *Klebsiella pneumoniae*. *J. Biol. Chem.* 267 8971–8976. 1577734

[B60] Vandemoortele G. Gevaert K. Eyckerman S. (2016). Proteomics in the genome engineering era. *Proteomics* 16 177–187. 26510734 10.1002/pmic.201500262

[B61] Wang W. Gu W. Ding Z. Ren Y. Chen J. Hou Y. (2005). A novel spiroplasma pathogen causing systemic infection in the crayfish *Procambarus clarkii* (Crustacea: Decapod), in China. *FEMS Microbiol. Lett.* 249 131–137. 10.1016/j.femsle.2005.06.005 16000238

[B62] Wang W. Gu W. Gasparich G. E. Bi K. Ou J. Meng Q. (2011). *Spiroplasma eriocheiris* sp. nov., associated with mortality in the Chinese mitten crab, *Eriocheir sinensis*. *Int. J. Syst. Evol. Microbiol.* 61 703–708. 10.1099/ijs.0.020529-0 20418415

[B63] Wang W. Wen B. Gasparich G. E. Zhu N. Rong L. Chen J. (2004). A spiroplasma associated with tremor disease in the Chinese mitten crab (*Eriocheir sinensis*). *Microbiology* 150(Pt 9), 3035–3040. 10.1099/mic.0.26664-0 15347761

[B64] Waterworth S. C. Flórez L. V. Rees E. R. Hertweck C. Kaltenpoth M. Kwan J. C. (2020). Horizontal gene transfer to a defensive symbiont with a reduced genome in a multipartite beetle microbiome. *mBio* 11:e02430-19. 10.1128/mBio.02430-19 32098813 PMC7042692

[B65] Watts T. Haselkorn T. S. Moran N. A. Markow T. A. (2009). Variable incidence of Spiroplasma infections in natural populations of *Drosophila* species. *PLoS One* 4:e5703. 10.1371/journal.pone.0005703 19492088 PMC2683927

[B66] Westberg J. Persson A. Holmberg A. Goesmann A. Lundeberg J. Johansson K. E. (2004). The genome sequence of *Mycoplasma mycoides* subsp. *mycoides* SC type strain PG1T, the causative agent of contagious bovine pleuropneumonia (CBPP). *Genome Res.* 14 221–227. 10.1101/gr.1673304 14762060 PMC327097

[B67] Xu B. Liu L. Song G. (2021). Functions and regulation of translation elongation factors. *Front. Mol. Biosci.* 8:816398. 10.3389/fmolb.2021.816398 35127825 PMC8807479

[B68] Yu J. Wayadande A. C. Fletcher J. (2000). *Spiroplasma citri* surface protein P89 implicated in adhesion to cells of the vector circulifer tenellus. *Phytopathology* 90 716–722. 10.1094/PHYTO.2000.90.7.716 18944490

